# Epidemiology As a Tool to Reveal Inequalities in Breast Cancer Care

**DOI:** 10.1371/journal.pmed.0030048

**Published:** 2006-01-17

**Authors:** Eduardo L Franco

## Abstract

Eduardo Franco discusses a new Swedish study that demonstrates systemic age-related disparities in access to breast cancer screening and care.

*“There is something in age that ever, even in its own despite, must be venerable, must create respect—and to have it ill treated, is to me worse, more cruel and wicked than anything on earth.”*

—Frances Burney, 1768

One of the noblest uses of epidemiology is the investigation of sources of inequalities in health outcomes. Research on prognostic factors of cancer survival fulfills this mission and is part of the expanded purview of cancer epidemiology. This type of research is best done in countries that are served by established tumour registries with efficient linkage to administrative databases that include patients' records from screening programs and hospital data on diagnoses and clinical outcomes. Such countries enjoy the benefits of a cancer surveillance system that covers the entire spectrum from primary to tertiary cancer prevention.

In this issue of *PLoS Medicine*, Eaker and colleagues describe a simple yet long-ranging observational study of breast cancer survival that uses Sweden's much-admired cancer surveillance system [1]. The authors conducted a population-based study that encompassed an entire health region to test the hypothesis that possible differences in diagnosis and management of breast cancer in older women would have an impact on survival. They studied more than 9,000 patients aged 50–84 years with breast cancer diagnosed between 1992 and 2002, calculating 5-year relative survival and related statistics that contrasted women aged 70–84 years with those who were 50–69 years at the time of diagnosis. Overall, the 5-year relative survival was 13% lower for those aged 70–84 years as compared with patients 50–69 years of age. The disparity in survival was greatest for those with disease stages IIB–III and for those whose tumour was not staged.

## How Can the Age-Related Prognostic Effect Be Explained?

Figure 1 shows a schematic of the underlying prognostic pathway model that was entertained by the authors. Having established that the distal, remote prognostic effect due to advanced age was real, they hypothesized that the association would be mediated largely by the intermediate variables shown in the model. The authors serendipitously explored the heterogeneity in their dataset that originated from the existence in their chosen health-care region of counties with more liberal policies that extend screening opportunity to women outside of the 50–69 year age range that was adopted by the Swedish health system. Measures of disease burden (tumour size and lymph node involvement) and markers of histological severity and hormonal responsiveness were available to the authors and formed one set of covariates, which they controlled for via stratification or regression model adjustment. Management and treatment variables, such as type and intent of surgery, radiation treatment, and chemotherapy (including tamoxifen use) were available as well. The authors used local clinical guidelines to judge whether or not the quality and quantity of treatment received via the three modalities were considered adequate and consonant with standard practices. They computed a low-treatment activity index specific for each stage grouping that subsumed the overall extent of suboptimal management and treatment decisions that may have affected the patient's prognosis (the central factor in Figure 1).

**Figure 1:**
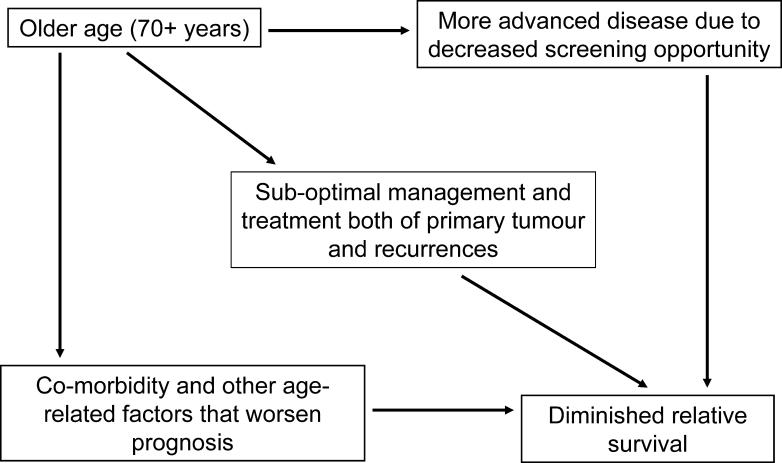
Model of Prognostic Pathway That Explains the Lower Breast Cancer Survival Expectation in Women Older than 70 Years The remote, distal prognostic effect is mediated via three intermediate, proximal determinants of clinical outcome. Ideally, only comorbidity and other age-related factors that are beyond the reach of modern health care should be operative. Unfortunately, decreased access to screening and to optimal treatment and management tend to further increase the disparity in survival between young and old women with breast cancer.

To test the empirical validity of the model in Figure 1, they first demonstrated that the top connecting relations (old age → advanced disease; old age → undertreatment) were real. Older women had fewer instances of screening-detected cancers, had larger tumours, had fewer nodes examined, and underwent radiotherapy or chemotherapy less frequently than younger women. Final proof that these imbalances in disease burden and treatment decisions were the actual mediators of the disadvantage in prognosis requires that the regression models relating age and relative mortality incorporate adjustment for diagnostic and tumour characteristics and treatment. If the remote prognostic relation is truly mediated by the latter variables, then the magnitude of the statistical effect (measured as relative excess mortality contrasting patients 70–84 years with those 50–69 years) would be diminished upon adjustment for the putatively intermediate covariates.

In fact, this is what the authors observed: the crude excess proportional mortality, which ranged between 43% and 83% for stages IIB, III, and unstaged patients, decreased to –9% to 26% after full adjustment for the intermediate variables (shown in Table 5 of [1]). Moreover, the statistical significance of the observed excess mortality disappeared after adjustment. In conclusion, the authors had elegantly demonstrated through clever use of regression modeling and mediated analysis [2,3] of breast cancer surveillance data that most of the original survival disadvantage was attributable to the biases against older women in access to health care.

## Lessons from a Scientific Standpoint

Unlike basic science disciplines, where the discovery process has more external cues, the reward for the pursuit of epidemiological findings exists exclusively from an intellectual perspective. The “eureka” factor in Eaker et al.'s study [1] comes from the simple realization that the adjusted estimates of excess mortality were lower than those seen in the univariate models, which implies that the original crude estimate reflected mostly the prognostic effect of the intermediate factors shown in Figure 1. The mediated analysis that they conducted is akin to the true experimentation and probing that is typical of laboratory-based research [3]. They formulated a hypothesis and verified it with crisp deductive logic by probing relations in an underlying model. The relevance of the conclusion cannot be overemphasized: had we eliminated the age-related systemic disparities in access to screening and cancer care, the substantial survival disadvantage of older breast cancer patients would have disappeared completely. As an epidemiologist, I cannot find a better use for an observational study.

## Lessons from a Public-Health Perspective

Eaker and colleagues conducted their observational study in a Swedish population, a country that enjoys one of the most progressive health-care systems in the world. Cancer screening is the right of all citizens, and ability to pay is not a determinant of access to oncological care in Sweden. Yet, local variations in practice do exist, largely due to policy decisions stemming from how different Swedish jurisdictions interpret the evidence base on the cost-effectiveness of screening and treatment modalities. Cancer screening, management, and treatment compete for funds with other pressing health-care needs.

Sweden does not come to mind when one ponders the typical dilemma of making funding decisions to prioritize health-care expenditures. Nevertheless, countries that can afford to sustain central planning and policy making in cancer control tend to favour providing the greatest benefit for the ones at highest risk and for the lowest possible cost. Given the low remaining life expectancy among the elderly and nearly complete exclusion of older patients from clinical trials, most countries have little incentive to implement health-promotion policies that do not overtly neglect older people. As the authors so poignantly concluded, this “is a very distressing finding, since around 30% of all breast cancer patients are above the age of 70.” The question that is left for all of us—not only those with careers devoted to cancer control—to answer is: to what extent do we as a society want to continue to assign lesser importance to our elderly when formulating health policies and research priorities?
